# Respiratory Function in Healthy Taiwanese Infants: Tidal Breathing Analysis, Passive Mechanics, and Tidal Forced Expiration

**DOI:** 10.1371/journal.pone.0142797

**Published:** 2015-11-11

**Authors:** Shen-Hao Lai, Sui-Ling Liao, Tsung-Chieh Yao, Ming-Han Tsai, Man-Chin Hua, Kuo-Wei Yeh, Jing-Long Huang

**Affiliations:** 1 Department of Pediatrics, Chang Gung Memorial Hospital Chang Gung University, Taoyuan, Taiwan; 2 Department of Pediatrics, Chang Gung University, Taoyuan, Taiwan; 3 Department of Pediatrics, Chang Gung Memorial Hospital and Chang Gung University, Keelung, Taiwan; 4 Prediction of Allergies in Taiwanese Children (PATCH) cohort study, Keelung, Taiwan; University of California, Merced, UNITED STATES

## Abstract

**Background:**

Although infant lung function (ILF) testing is widely practiced in developed Western countries it is not typically performed in Eastern countries, and lung measurements are scarce for Asian infants. Therefore, this study aimed to establish normal reference values for Taiwanese infants.

**Materials and Methods:**

Full-term infants without any chronic diseases and major anomalies were enrolled in the Prediction of Allergies in Taiwanese Children (PATCH) cohort study. Detailed medical data, such as body weight and length, birth history, and histories of previous illness and hospitalization were recorded. Lung function measurements such as analysis of tidal breathing, passive respiratory mechanics, and forced tidal expiratory flow-volume curves were obtained through Jaeger Masterscreen BabyBody Paediatrics System. Multiple linear analyses were performed to determine various parameters of the lung function tests.

**Results:**

ILF test parameters were collected from 126 infants, and 189 tests were performed. The results revealed that the ratio of time to peak expiratory flow to total expiratory time, the ratio of volume to peak expiratory flow to total expiratory volume, and the ratio of inspiratory time to total respiratory time remained relatively constant despite differences in age. However, body length is the strongest independent variable influencing tidal volume, respiratory rate, resistance, compliance, and maximal expiratory flow at functional residual capacity.

**Conclusion:**

According to our review of relevant literature, this is the first study to establish a reference data of ILF tests in the Asian population. This study provided reference values and regression equations for several variables of lung function measurements in healthy infants aged less than 2 years. With these race-specific reference data, ILF can more precisely and efficiently diagnose respiratory diseases in infants of Chinese ethnicity.

## Introduction

Infant lung function (ILF) has been widely used for research and clinical practices in Western countries. ILF testing is useful in the early diagnosis of lung diseases [1] and in serial monitoring of disease progression [2,3]. In addition, routine implementation of ILF may improve the efficacy of therapeutic interventions [4]. In research, it can be used to determine the effects of environmental or inflammatory insults during perinatal life. The American Thoracic Society/European Respiratory Society (ATS/ERS) task force has developed clinical practice guidelines for ILF [5–7]; moreover, multiple reference equations have been developed [8–11]. However, the general applicability of these reference equations remains disputed in settings where equipment- [12] and ethnicity-specific differences might occur [13,14]. Until date, no reference data are available for infants of Asian ethnicity. Establishing equipment- and ethnicity-specific reference equations of IFT for the Asian population should improve interpretation of both clinical and research studies.

The aim of this study was to establish a reference data for ILF tests in Taiwanese children during the first 2 years of life. Measurements included tidal breathing analysis, passive respiratory mechanics, and forced tidal expiratory flow-volume curves. Moreover, prediction equations that could be expressed as Z scores were developed after considering sex, age, body weight, and body height.

## Materials and Methods

### Participants

Data were obtained from an ongoing prospective birth cohort study called the Prediction of Allergy in Taiwanese Children (PATCH). PATCH is an unselected, population-based study investigating the risk factors for immune-related and allergic diseases in children in Northern Taiwan. Detailed descriptions of subjects recruitment and data collection have been reported previously [15–17]. The study was approved by the Chang Gung Ethics Committee, and written informed consents were obtained from the parents or legal guardians of the infants. All participant’s records and clinical information were anonymized and deidentified before analysis. Children born prematurely (gestational age <36 weeks), those with major birth defects or congenital structural anomalies of the upper airway, those who were hemodynamically unstable, and those with a history of severe lower airway infection with intensive care unit admission were excluded from the study.

### Lung Function Tests

Measurements were performed in healthy infants without respiratory tract infection for at least 3 weeks. Before the tests, body weight was measured and crown-heel length was obtained on measuring board. Subsequently, the infants were sedated with oral chloral hydrate (50–75 mg/kg), and placed in the supine position, with the neck mildly extended. ILF tests were performed through Jaeger Masterscreen BabyBody Paediatrics System (CareFusion, Hoechberg, German). The equipment conforms with the of ATS/ERS recommendations [5–7,18].

#### Tidal breathing analysis

An epoch of at least 20 stable tidal breaths was collected after the breathing pattern of the infants stabilized. The following parameters were collected from the flow and volume signals for data analysis: total respiratory time (Tt), inspiratory time (Ti), total expiratory time (T_E_), total expiratory volume (Ve), tidal volume (Vt), peak expiratory flow (PEF), time and exhaled volume to peak expiratory flow (T_PEF_ and V_PEF_), respiratory rate (RR), and minute ventilation (MV). All procedures and data acquisition were performed following the guidelines of the ATS/ERS task force [5]. In house precision testing of Vt showed intra-test coefficients of variation (CV) was 7.5 ± 5.8%.

#### Respiratory mechanics

Passive respiratory mechanics were measured using the single occlusion technique, in accordance with the ATS/ERS recommendations [6]. Briefly, single occlusion of the airway at the end of tidal inspiration induces a Hering—Breuer reflex with subsequent relaxation of the respiratory muscle, followed by rapid equilibration being reached during period of no flow. When pressure reaches equilibrium throughout the airway, the airway opening pressure (*P*_ao_) will accurately represents the alveolar pressure of the examined subject. *P*_ao_ in turn represents the summed elastic recoil pressure of lung and chest wall during period of muscle relaxation. The passive exhalation flow-volume loop was obtained on valve opening. The expiratory flow-volume curve and *P*_ao_ can subsequently be related to changes in volume and flow, enabling calculation of the expiratory time constant (*τ*_rs_), resistance (Rrs), and compliance (Crs). At least three technically acceptable end-inspiratory occlusions were used to assess the mean *τ*_rs_, Rrs, and Crs. The intra-test CV of Rrs and Crs was 9.8 ± 3.7% and 7.7 ± 5.8%, respectively

#### Forced tidal expiration

Forced tidal expiratory flow-volume curves were obtained through rapid thoracoabdominal compression (RTC) by following the ATS/ERS guidelines [7]. To summarize, RTC was performed using an inflatable jacket that covered the chest and abdomen but not the arms of the infant. At least five tidal breaths were sampled before the maneuver to accurately determine the point of functional residual capacity (FRC) in flow-volume curve using the last three tidal breathings. After the tidal breathing was stabilized, the forced expiratory maneuver was initiated from the end inspiration of a tidal breath. An initial jacket pressure of 3 kPa was applied, and the procedure was repeated by increasing the jacket pressure in 1–2 kPa intervals until flow limitation was achieved. The maximal expiratory flow at FRC (VmaxFRC) was subsequently computed and reported as the highest of at least three technically acceptable curves (generally within 10% difference). The intra-test CV of VmaxFRC showed 5.0 ± 3.2%. Fifty-five children were further enrolled in a study of short-term repeatability. We analyzed the consistency between two measurements of VmaxFRC, conducted 15 minutes apart.

### Statistical Analysis

Normal probability plots were used to assess whether the normality assumption of Rrs, Crs, and VmaxFRC was appropriate. The results indicated that the assumption of normal distribution was reasonable for these parameters, therefore, parametric analyses were performed. The Spearman correlation analysis was performed to delineate the relationship between lung function parameters and demographic data of the subjects. A stepwise multiple regression was used to determine the characteristics that significantly improved the fraction of observed differences in the dependent variables. Subsequent development of the prediction equations was attained. Between-test repeatability of VmaxFRC was determined using Bland—Altman analysis. The relative coefficient of repeatability was calculated as 2*SD of the difference between two measurements setting as a ratio of the mean of the two measurements. P < 0.05 was considered significant. All statistical analyses were performed using IBM SPSS Statistics version 20 (Armonk, NY, USA).

## Results

We performed 189 tests on 126 infants (63 boys). The median age was 8 months (range, 5–26 months) and median body length was 73 cm (range, 65–95 cm). All but seven infants (who were born at 36 weeks gestation) were born at ≥37 weeks gestation. Table 1 summarized the demographic characteristics of participants. The number and success rate of lung function measurements performed on infants of various ages are listed in Table 2.

**Table 1:** Demographic characteristic of 189 lung function tests on 126 infants.

	n*	Mean (SD)	Range
Male, n (%)	126	63 (50%)	
Gestational age, weeks	126	38.4 (1.2)	36–41
Birth weight, kg	126	3.2 (0.4)	2.0–4.4
Birth weight, Z-score	126	-0.1 (0.8)	-2.3–2.4
Birth length, cm	126	50.2 (2.7)	33.0–56.0
Birth length, Z-score	126	-0.1 (0.8)	-2.3–2.4
Weight, Z-score^#^	189	-0.2 (1.0)	-2.6–2.0
Height, Z-score^#^	189	-0.1 (1.2)	-3.6–2.8
Maternal smoking during pregnancy, n (%)	126	3 (2.4%)	
Household smoking, n (%)	126	56 (45.2%)	

Data expressed as number (%) or mean (SD) unless otherwise stated.

* n described the number of participants or test occasions.

^#^ Body weight and height obtained during test occasion.

**Table 2:** Number and success rate of 189 lung function tests on 126 infants (50% male).

Age of test (number of test)	5–9 mo (102)	10–15 mo (53)	16–21 mo (22)	22–26 mo (12)
*Tidal breath analysis* *	102 (100)	53 (100)	22 (100)	12 (100)
*Passive mechanics* *	95 (93)	50 (94)	21 (95)	12 (100)
*Forced tidal expiration* *	91 (89)	48 (90)	21 (95)	10 (83)

* Test number of lung function exams (% of successful rate) was shown.

**Fig 1:**
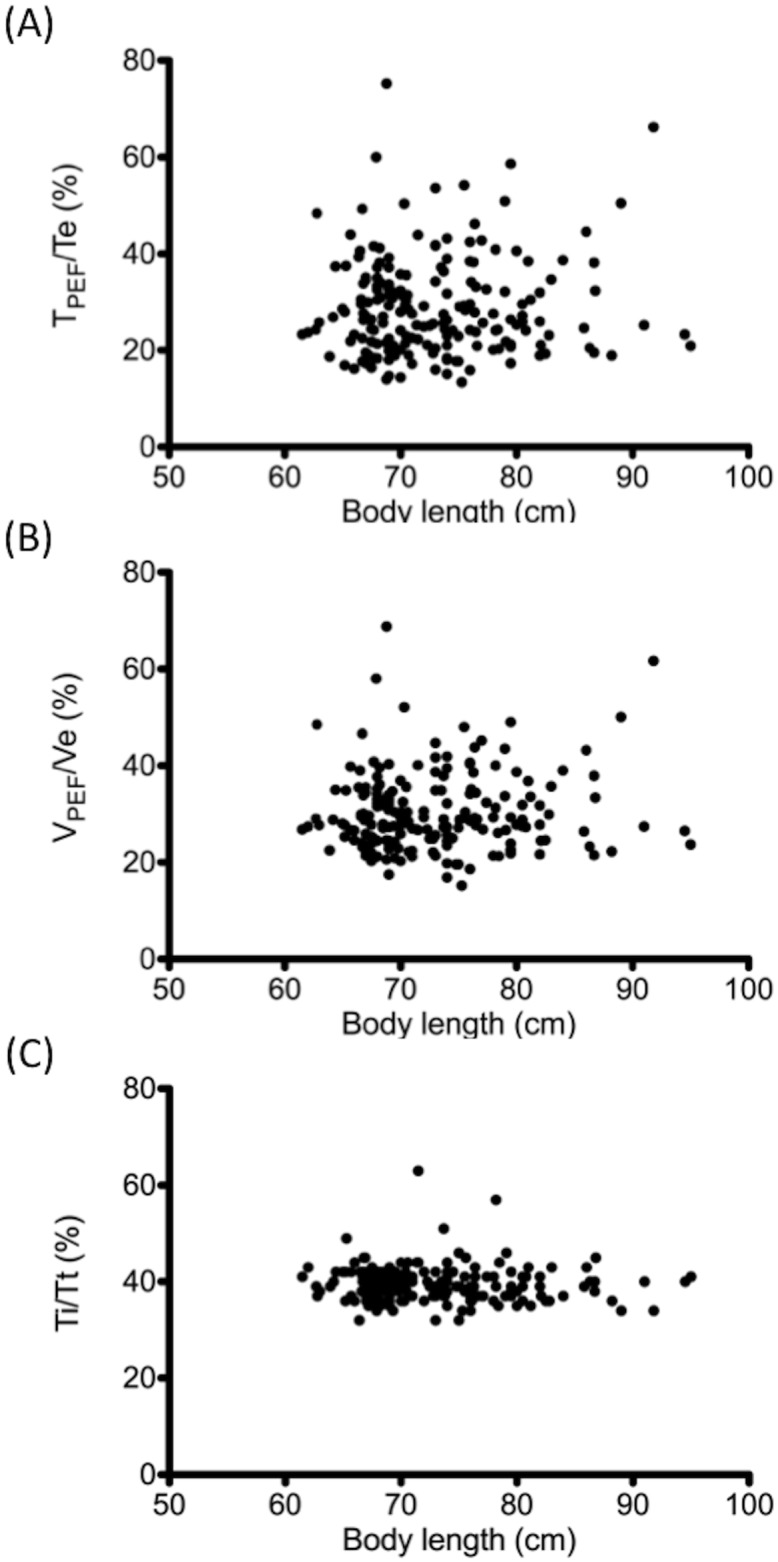
Parameters for tidal breathing analysis. Scatter plots of (A) T_PEF_/Te, (B) V_PEF_/Ve, and (C) Ti/Tt in tidal flow waveform are indicated against crown-heel length at the time of the test.

Various parameters of the tidal flow waveform analysis are summarized in Table 3. Mean flow ratios T_PEF_/Te, V_PEF_/Ve, and Ti/Tt remained relatively constant among the various age groups. Furthermore, scatter plots of these three parameters against crown-heel body length are depicted in Fig 1. No significant correlation was discovered between sex, body weight, and body length with these tidal flow waveform parameters.

**Table 3:** Tidal flow parameters, expressed as mean ± standard deviation, of infants in various age groups.

Age of test	Total	5–9 mo	10–15 mo	16–21 mo	22–26 mo
T_PEF_/Te *(%)	29 ± 10	29 ± 10	29 ± 10	28 ± 9	29 ± 14
V_PEF_/Ve ^†^(%)	30 ± 8	30 ± 7	31 ± 8	30 ± 8	31 ± 12
Ti/Tt ^‡^(%)	39 ± 3	40 ± 4	39 ± 4	39 ± 3	39 ± 3

* Ratio of time to PEF to total expiratory time;

^†^ ratio of volume to PEF to total expiratory volume;

^‡^ ratio of inspiratory time to total respiratory time.

**Fig 2:**
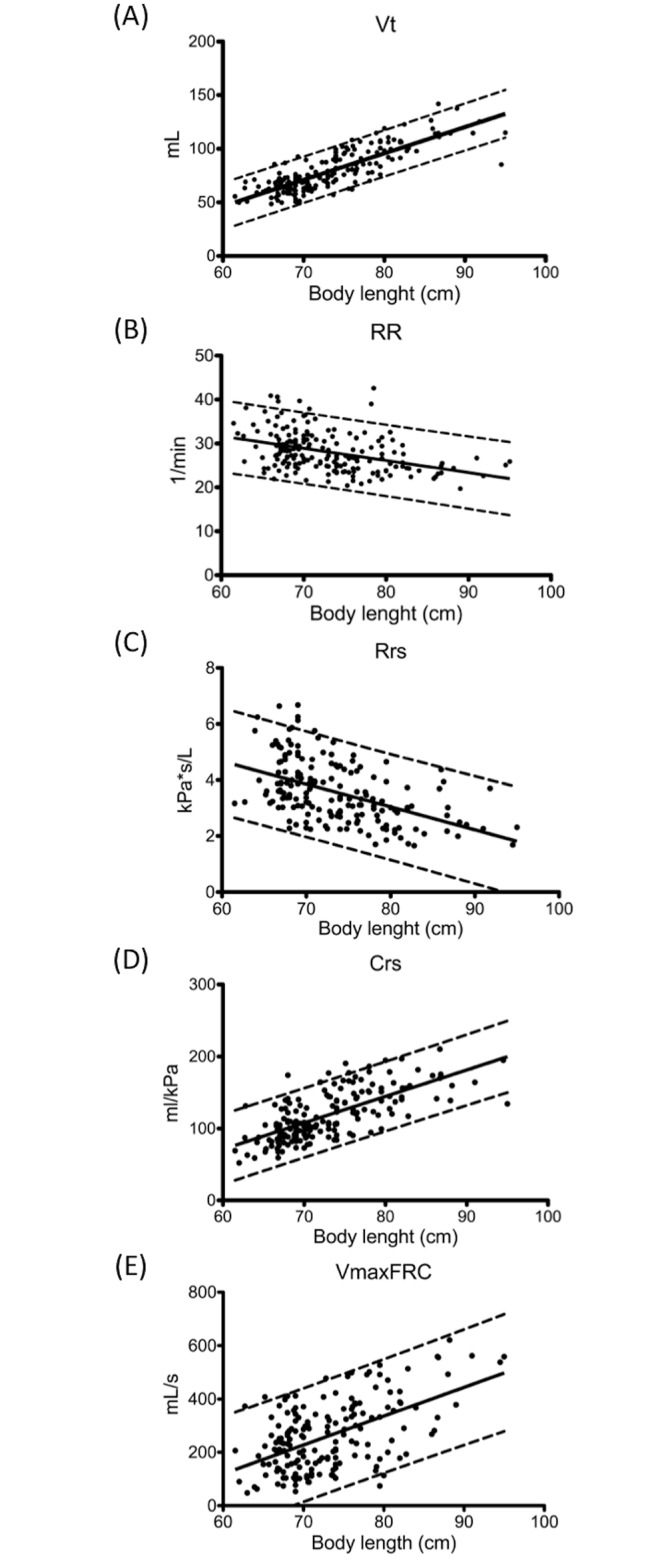
Regression curves of various parameters. Regression curves (means and 95% confidence bands) of various parameters including (A) tidal volume (Vt), (B) respiratory rate (RR), (C) resistance (Rrs), (D) compliance (Crs), and (E) maximal flow at functional residual capacity (VmaxFRC), plotted against crown-heel length at the time of the test.

In the stepwise multiple linear regression analysis (variables were age, body length, body weight, and sex), body length was the most significant independent variable associated with Vt, RR, Rrs, Crs, and VmaxFRC. The optimal powers of the regression equation relating the body length to Vt, RR, Rrs, Crs, and VmaxFRC were 15.6%, 68%, 23.7%, 57.3%, and 33.6%, respectively. No sex-related difference was observed in the slope and intercept of linear regression curves. Scatter plots and regression lines representing the mean and 95% confidence interval of the test results are depicted in Fig 2. Table 4 lists the sex-specific regression equations of these parameters. The standard deviations of the residual values were used to compute the z score of an observed value. In general, girls had higher coefficients of determination (R^2^) of the regression equation than did the boys.

**Table 4:** Regression equations of various parameters of infant lung function.

Variable		Coefficient*	p value for commonality with girls	SE of coefficient*	RSD	R^2^
***Vt*** ^†^						
**Total**	Constant	-101.392	-	8.919	10.444	0.680
	BL, cm	2.463	-	0.122		
Boys	Constant	-100.488	0.88	13.749	12.372	0.649
	BL, cm	2.464	0.79	0.185		
Girls	Constant	-97.617	-	11.537	9.193	0.706
	BL, cm	2.396	-	0.160		
***RR*** ^‡^						
**Total**	Constant	48.308	-	3.352	3.919	0.161
	BL, cm	-0.277	-	0.046		
Boys	Constant	46.766	0.72	4.654	4.188	0.152
	BL, cm	-0.259	0.77	0.063		
Girls	Constant	49.229		5.078	4.046	0.151
	BL, cm	-0.287		0.071		
***Rrs*** ^§^						
**Total**	Constant	9.668	-	0.816	0.957	0.246
	BL, cm	-0.083	-	0.011		
Boys	Constant	9.046	0.33	1.052	0.911	0.237
	BL, cm	-0.074	0.28	0.014		
Girls	Constant	10.949	-	1.309	0.992	0.280
	BL, cm	-0.102	-	0.018		
***Crs*** ^||^						
**Total**	Constant	-191.949	-	20.401	23.585	0.573
	BL, cm	4.270	-	0.279		
Boys	Constant	-187.195	0.29	31.237	25.060	0.544
	BL, cm	4.258	0.41	0.421		
Girls	Constant	-180.733	-	26.322	20.583	0.593
	BL, cm	4.060	-	0.365		
***VmaxFRC*** ^¶^						
**Total**	Constant	-595.497	-	92.690	104.519	0.340
	BL, cm	11.719	-	1.269		
Boys	Constant	-489.596	0.60	132.662	109.540	0.285
	BL, cm	10.310	0.60	1.791		
Girls	Constant	-722	-	133.266	98.746	0.395
	BL, cm	13.452	-	1.850		

BL, body length.

* All P < 0.001;

^†^ tidal volume;

^‡^ respiratory rate;

^§^ resistance;

^||^ compliance;

^¶^ maximal expiratory flow at functional residual capacity.

**Fig 3:**
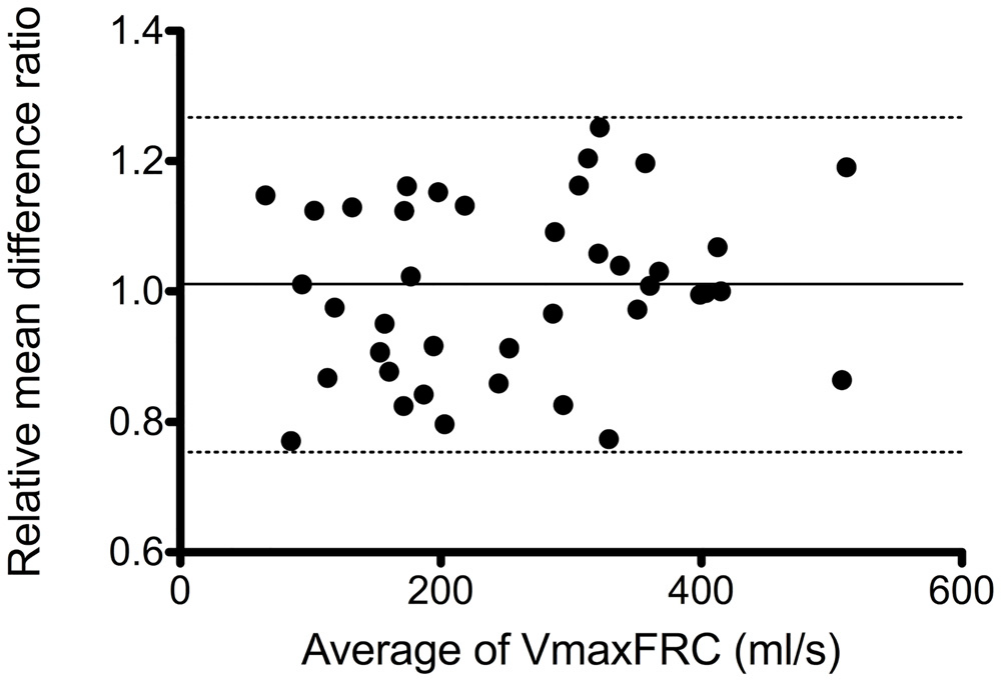
Short-term repeatability of VmaxFRC. Bland-Altman plot of the agreement in VmaxFRC is analyzed between two measurements sets 15 min apart. Data are plotted as the difference between two measurements versus the mean of the two measurements. Differences for individuals are shown as solid circles. The mean of the differences and the upper and lower limits of agreement are plotted as solid and dash lines respectively.

The between-test repeatability of paired VmaxFRC measurements for over a 15 min period were attempted in 55 children and were successful in 40 (17 boys). As shown in Fig 3, difference between successive tests was not related to the value of the mean of two measurements. The relative coefficient of repeatability equated to a mean relative difference (difference as a ratio of the mean of the two measurements) was 0.26, namely 26%.

## Discussion

According to our review of relevant literature, this study is the first to report ILF reference values and equations for Taiwanese children aged 5–26 months. Our results revealed that the values of the tidal flow waveform, including T_PEF_/Te, V_PEF_/Ve, and Ti/Tt, remained consistent among various age groups. However, in the regression equations of Rrs, Crs, and VmaxFRC, body length was found to be the most vital independent variable associated with these parameters. These reference values can enhance the accuracy and effectiveness of the clinical assessment of respiratory functions in children with Chinese ethnicity.

### Comparisons with the Literature Data

Due to differences in measuring equipment and questionable conformations to the ATS/ERS guidelines, it is difficult to compare our reference curves with previously published reference values. Besides, the coefficients of determination (R^2^) can be influenced by both the body-length range and number of participants. Nonetheless, comparisons with other published references were attempted for various parameters of ILF.

#### Tidal breathing analysis

Consistent with previous reports, the T_PEF_/Te and V_PEF_/Ve ratios obtained by our study remained remarkably constant from infancy to the preschool age, with these ratios ranging from 25% to 45% and 35% to 42%, respectively [11,19–23]. In most studies, tidal flow was measured during the awake or natural sleep state, nevertheless, the values measured during the sedated sleep state in our study yielded results comparable with those reported earlier.

#### Respiratory mechanics

**Fig 4:**
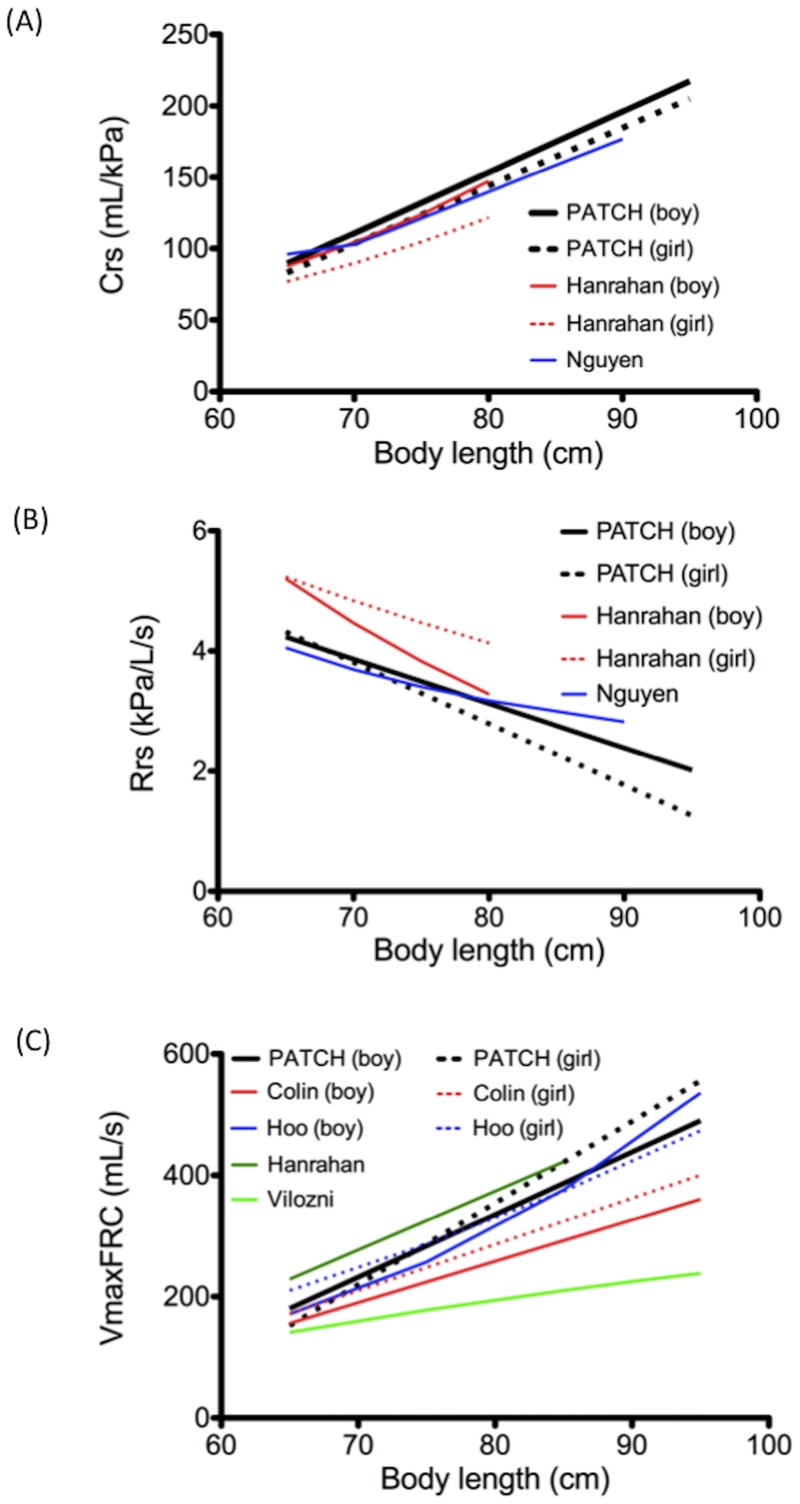
Comparison of various reference curves. Present regressions of (A) Crs, (B) Rrs, and (C) VmaxFRC in this study compared with previously published data.

Respiratory mechanics measured by the single occlusion technique were mostly performed in neonates; thus, limited data was available for older infants. [9,11,24], rendering respiratory mechanics a critical issue to be explored because reference values obtained from respiratory mechanics might differ with infant growth. Accordingly, similar to the observation of Hanrahan et al. [24], we observed that body length influenced the test results; body length was related to decreasing Rrs and increasing Crs. The scarce respiratory mechanics reference data currently available was only for infants with body lengths ranging from 65 to 75 cm [25,26]. The results of our study provide a wider range of data for infants with body length exceeding 90 cm, which can be used for future reference. The Crs obtained from this study was similar to that reported by Hanrahan et al. (Fig 4A), but the Rrs was lower (Fig 4B). However, reference curves of Crs and Rrs of the study were all comparable with the recent reference curves of Nguyen et al (Fig 4A and 4B) [23]. Since the work of Hanrahan et al. was performed before the publication of ATS/ERS guidelines, it was difficult to compare to the difference with our results.

#### Forced tidal expiration

Several results of VmaxFRC in healthy Caucasian infants have been published recently [10,27,28]. Our reference data of VmaxFRC are comparable with those reported by Hanrahan and Hoo; however, the values from our study were considerably higher than those reported by Colin (Fig 4C), possibly because of the distinct methodologies used for the tests. The maximum pressure of the inflatable jacket used by Colin et al. was only 10 kPa [28]; however, the jacket pressure that we used for examining infants older than 12 months often exceeded 10 kPa. In the study, the means (and 95% confidence intervals) of jacket pressure in different age group were as below: 5–9 mo, 8.7 (8.4–9.0) kPa; 10–15 mo, 10.6 (10.2–11.0) kPa; 16–21 mo, 11.7 (11.2-–12.2) kPa; 22–26 mo, 12.7 (11.6-–13.8) kPa. According to the ATS/ERS guidelines [7], jacket pressure was increased in 1–2 kPa increments until the optimal pressure was achieved; no upper limit of jacket pressure is mentioned in the guidelines. Thus, the differences in the applied pressure may cause the difference in the results. Recently, a much lower reference curve was reported by Vilozni’s report [29] (Fig 4C). Difference of measuring equipment might contribute to the disparity.

### Sedation of Infant Lung Function Testing

The ATS/ERS guidelines do not recommend using specific sedatives for ILF testing. Chloral hydrate has been widely prescribed in an unapproved off-label manner for many pediatric clinical procedures. In addition, it has been the preferred medication for ILF testing at multiple centers worldwide. Although alternate sedatives, such as midazolam or ketamine, have been tried, the use of these alternatives has been reported to skew test results [30,31]. Our experience has shown that chloral hydrate is a relatively effective and safe sedative for infants. Vomiting, which occurred in approximately 7% (14/189) of the examinations, appeared to be the most common adverse effect.

### Reliability of VmaxFRC

Spirometry has long been standardized for children older than 6 years. However, partial flow-volume maneuvers, such as tidal RTC, had not been standardized in infants until 2000 [7]. The measurement of VmaxFRC is based on the determination of FRC before tidal RTC. A strong positive relationship exists between VmaxFRC and the extent to which FRC is dynamically elevated above the end-expiratory level [32]. However, FRC is not a stable landmark, particularly in young infants in whom the end-expiratory level may be dynamically high. Moreover, flow limitation is occasionally difficult to achieve in healthy infants [33]. Thus, both the aspects can contribute to the high variability in VmaxFRC measurement. In our study, the Bland—Altman analysis revealed that the mean percentage of bias was 0.5%, and 95% of the upper and lower limits of agreement were 74% and 126%, respectively suggesting that intra-individual variability was within the acceptable range.

Ideally, ILF should be performed using methods allowing full inflation of the lung to total capacity and subsequent exhalation to residual volume. However, this ideal condition requires endotracheal intubation and general anesthesia, a procedure that cannot be widely applied to the general population. Recently, raised-volume RTC enables examiners to obtain a near-full-inflation flow-volume curve [34]. Although endotracheal intubation and general anesthesia is not required in performing the examination, however, the test is time intensive and requires more complex equipment. Thus, to date, VmaxFRC is commonly investigated using the tidal RTC technique during clinical practice because of its easy applicability and accessibility.

### Limitations in the Interpretation of Reference Values

Although the present study provides the largest local normative reference values of ILF, some concerns limit its general applicability in clinical practice. First, as mentioned earlier, because of significant variations in the reference values among different studies and populations (Fig 3), a comparison becomes difficult when only the mean predicted curve is considered, and it becomes more difficult if variability is not accounted for. Hence, a comparison of the Z scores rather than that of the absolute value is suggested when evaluating the lung function status of a subject. Second, population selection bias may be present because the majority of the participants from this study live in Keelung City, a city in Northern Taiwan; this can affect the generalizability of the reference values. However, after comparing with local normal population [35], the Z-scores of demographic data of study population all approximated zero (Table 1). The results revealed that our study population was similar to the normal local population. Third, the number of participants with body-length >85cm was relatively few (15 children). It would cause wider range of upper and lower 95% confidence limit. Care should be taken during application of the reference equation in children >85cm in length.

Furthermore, defining what constitutes a “normal healthy” infant is difficult because this study aimed to develop reference values for ILF tests. We applied minimal exclusion criteria to eliminate children with obvious respiratory tract anomalies and infections and those suffering from illness that might affect the test results. However, although infants with a history of smoke exposure generally appeared “normal”, some reports have suggested that exposure to cigarette smoke during the prenatal and postnatal period might adversely affect the respiratory function of infants [36,37]. Nevertheless, we did not exclude children with smoke exposure, because such an exclusion criterion raised the question of how representative the sample was of a population of healthy young children. Only three mothers (2.4%) reported smoking during pregnancy in this study. The proportion of prenatal smoke exposure was comparable with previous local report (3.1%) [38]. Furthermore, in our unpublished data, no significant differences in various parameters were observed between infants with and without smoke exposure. In addition, this problem was compounded because smoke-exposure history was self-reported, thus lacking a quantitative measure. Thus, the accuracy of the normative reference values obtained in our study remains debatable.

Finally, depending on the published equations for interpreting ILF data, particularly for the forced expiratory flow-volume curve, may lead to misinterpretation in both clinical and research settings. Using modern commercially available equipment, the result of lung function testing in healthy infants in Lum’s study was considerably different from the published data [12]. Therefore, future research is necessary to establish equipment-specific data for meaningful interpretation of ILF in healthy infants.

## Conclusion

This study presented reference values of ILF for Taiwanese infants with Chinese ethnicity. We reported the mean values and standard deviations of various lung function parameters, including the tidal breathings, passive respiratory mechanics, and forced tidal expiration. Moreover, we established sex-specific prediction equations of specific parameters for infants aged 5–26 months within the body-length range of 65–95 cm. These race-specific reference data can enhance the accuracy and effectiveness of the clinical application of ILF for diagnosing respiratory diseases in infants of Chinese ethnicity.
